# Frailty in Nonalcoholic Fatty Liver Cirrhosis: A Comparison with Alcoholic Cirrhosis, Risk Patterns, and Impact on Prognosis

**DOI:** 10.1155/2021/5576531

**Published:** 2021-05-21

**Authors:** Lubomir Skladany, Pavol Molcan, Jana Vnencakova, Petra Vrbova, Michal Kukla, Lukas Laffers, Tomas Koller

**Affiliations:** ^1^Department of Hepatology, Gastroenterology, and Transplantation (HEGITO), 2^nd^ Department of Medicine, Slovak Medical University, FD Roosevelt Faculty Hospital, Nám. L. Svobodu 1, Banska Bystrica 97517, Slovakia; ^2^Gastroenterology and Hepatology Subdivision, 5^th^ Department of Medicine, Comenius University Faculty of Medicine, University Hospital Bratislava, Ruzinovska 6, Bratislava 82606, Slovakia; ^3^Department of Internal Medicine and Geriatrics and Department of Endoscopy, Jagiellonian University Medical College and University Hospital in Cracow, Jakubowskiego 2, Kraków 30-688, Poland; ^4^Department of Mathematics, Faculty of Natural Sciences, Matej Bel University, Tajovského 40, Banská Bystrica 97401, Slovakia

## Abstract

**Background:**

Physical frailty increases susceptibility to stressors and predicts adverse outcomes of cirrhosis. Data on disease course in different etiologies are scarce, so we aimed to compare the prevalence and risk factors of frailty and its impact on prognosis in nonalcoholic fatty liver (NAFLD) and alcoholic (ALD) cirrhosis. *Patients and Methods*. Cirrhosis registry RH7 operates since 2014 and includes hospitalized patients with decompensated cirrhosis, pre-LT evaluation, or curable hepatocellular carcinoma (HCC). From the RH7, we identified 280 ALD and 105 NAFLD patients with at least 6 months of follow-up.

**Results:**

Patients with NAFLD compared with ALD were older and had a higher proportion of females, higher body mass index (BMI) and mid-arm circumference (MAC), lower MELD score, CRP, and lower proportion of refractory ascites. The liver frailty index did not differ, and the prevalence of HCC was higher (17.1 vs. 6.8%, *p*=0.002). Age, sex, serum albumin, and C-reactive protein (CRP) were independent predictors of frailty. In NAFLD, frailty was also associated with BMI and MAC and in ALD, with the MELD score. The Cox model adjusted for age, sex, MELD, CRP, HCC, and LFI showed that NAFLD patients had higher all-cause mortality (HR = 1.88 95% CI 1.32–2.67, *p* < 0.001) and were more sensitive to the increase in LFI (HR = 1.51, 95% CI 1.05–2.2).

**Conclusion:**

Patients with NAFLD cirrhosis had a comparable prevalence of frailty compared to ALD. Although prognostic indices showed less advanced disease, NAFLD patients were more sensitive to frailty, which reflected their higher overall disease burden and led to higher all-cause mortality.

## 1. Introduction

Pandemics of inactivity and sarcopenic obesity rapidly increase the global burden of NAFLD [1, 2], which is estimated at 25% and is expected to increase substantially until 2030 [3, 4]. To attract more attention of the general public, it has been recently proposed to rename nonalcoholic fatty liver disease (NAFLD) to “metabolic-associated fatty liver disease” (MAFLD) [5, 6]. Slovakia, with 349 cases of decompensated advanced chronic liver disease (ACLD) per 100,000 inhabitants, ranks number one in the world. The leading cause is alcoholic liver disease (ALD), and the fastest-growing cause is NAFLD [7]. Sarcopenia in NAFLD compared to other cirrhosis etiologies lies higher upstream in the disease pathophysiology. Several reports have highlighted the negative impact of sarcopenia in NAFLD and ACLD [8–19]. Although diagnosing sarcopenia according to the European Working Group on Sarcopenia in Older People (EWGSOP2) consensus is indispensable in academic research, it is less convenient in real-life hepatology practice [20–30]. In contrast, a simple bedside evaluation of muscle strength in ACLD is also predictive of adverse outcomes [31]. The concept of physical frailty, which is defined by the loss of physiologic reserve and increased susceptibility to stressors, was recently translated from geriatrics to hepatology [32–34]. The functional domains that are best validated for quantifying physical frailty are hand grip strength, chair stand speed, gait speed, and balance time. Physical frailty is an independent predictor of prognosis in ACLD along with a model for end-stage liver disease (MELD) and predicts a range of adverse outcomes in liver transplant (LT) candidates and hospitalized patients [35–41]. The prevalence of frailty in patients with ACLD is estimated at 20–35%, and no difference was found between the sexes [35, 40, 42, 43]. Although some reports have suggested a higher prevalence of frailty in NAFLD cirrhosis, few studies are addressing this issue [40, 43]. The aim of our study was therefore to compare the prevalence of physical frailty, risk factors for its occurrence, and its impact on the prognosis of patients with NAFLD and alcoholic cirrhosis.

## 2. Patients and Methods

The HEGITO7 registry (RH7) operates in the Department of Hepatology, Gastroenterology, and Transplantation (HEGITO), since 2014. The entry criteria for the registry are as follows: signed informed consent, ACLD requiring hospitalization, and event of cirrhosis decompensation, or evaluation for liver transplantation (LT), or hospitalization for hepatocellular carcinoma (HCC) within the Milan criteria. The registry does not include patients hospitalized for elective procedures, or terminal stages of ACLD or HCC, or with a severely limited life expectancy. The registry contains the date of index hospitalization, basic demographics, medical history, cirrhosis etiology and complications (refractory ascites, RA), body mass index (BMI), hand grip strength (HGS, in kg, using the dynamometer Kern MAP80), mid-arm circumference (MAC, in cm), and tricipital skinfold (in mm, using Harpenden type caliper Somet). During hospitalization, laboratory parameters are recorded (blood count, inflammatory, and synthetic liver function markers). MELD-Na score (further referred to as MELD), Child-Pugh-Turcotte score, and time are needed to complete the number connection test (25 numbers). Since 2017, all patients have been evaluated for functional status by measuring the time required to five chair stands without the help of hands and balance time in three feet positions (parallel, tandem, and semitandem). From the measured parameters, we calculated the liver frailty index (LFI) using a web-based online calculator (https://liverfrailtyindex.ucsf.edu). Since 2019, data from other hospitals in the country are being added to the registry using the same protocol.

For the present study, data from two hospitals were available for analysis. The entry criteria were as follows: patients in the RH7 registry who had NAFLD or ALD, which was considered a salient cause of ALCD, complete data on functional parameters at baseline, and follow-up of at least 6 months.

All procedures involving human participants have been carried out according to the ethical standards of the institutional research committee, including the 1964 Helsinki Declaration and its later amendments (http://www.wma.net) or comparable ethical standards. The reported clinical and research activities are consistent with the Principles of the Declaration of Istanbul, as outlined in the Declaration of Istanbul on Organ Trafficking and Transplant Tourism. All patients signed informed consent before enrolment into the registry, and data acquisition was approved by the local ethics committee: Etická komisia Fakultnej Nemocnice s Poliklinikou F. D. Roosevelta (in English: Ethics Committee of the Faculty Hospital F.D. Roosevelt), address: Etická komisia, FNsP FD Roosevelta, Nám. L. Svobodu 1, 975 17 Banská Bystrica, Slovakia, on May 21^st^, 2014.

Due to nonnormal data distribution, numerical parameters are presented as medians and 25–75 percentiles, while proportions are given as numbers and percentages. For comparison of numerical parameters and proportions, we used the Mann–Whitney and chi-square tests, respectively. Missing values were treated as missing and were not accounted for in statistical models. Definition of frailty was adopted according to Lai et al. 2017 (LFI > 4.5) and by calculating the 80^th^ percentile of LFI in our entire cohort of 385 patients (LFI > 5.2). To compare factors associated with frailty between the two etiologies, we constructed linear and multivariable models. Dependent variables were either numerical LFI or categorical frailty (LFI > 4.5). In either case, we used a backward regression model to select covariates independently associated with frailty according to the *p* value < 0.05.

**Figure 1:**
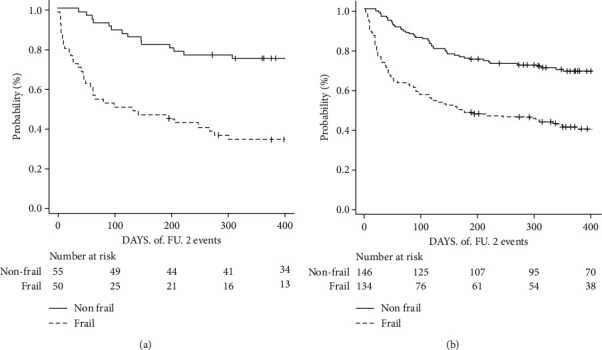
Kaplan–Maier transplant-free survival probability curves and a log-rank test by frailty status, solid line LFI ≤ 4.5, and dotted line LFI > 4.5, in alcoholic cirrhosis (right pane) and NAFLD cirrhosis (left pane), ^*∗*^*p* < 0.0001. (a) NAFLD cirrhosis^*∗*^. (b) ALD cirrhosis.

**Figure 2:**
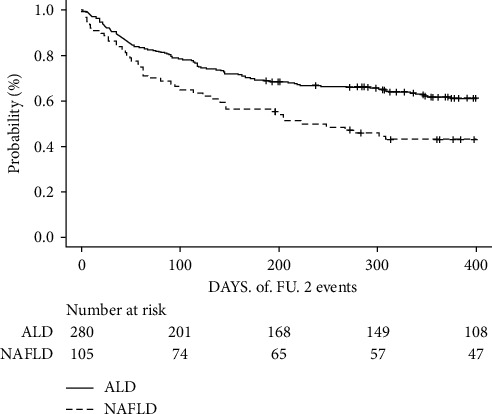
Adjusted Cox model for the probability of transplant-free survival in NAFLD cirrhosis (dotted line) and ALD cirrhosis (solid line). NAFLD HR = 1.9 (95% CI 1.31–2.7).

**Figure 3:**
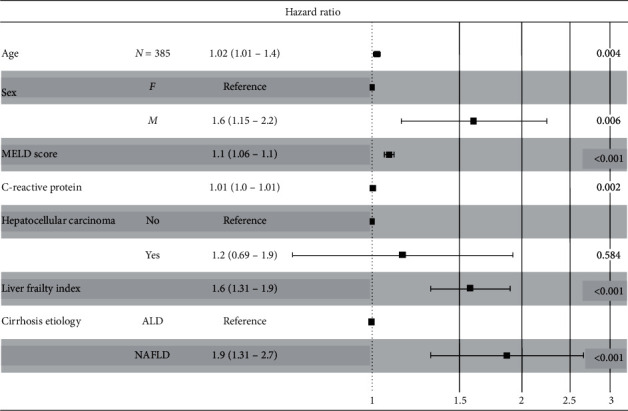
Forest plot of the adjusted Cox model for the predictors of death or LT from the RH7 cirrhosis registry (ALD = 280, NAFLD = 105), events *n* = 188, global *p* value (Log-rank) <0.0001, AIC = 1072, and concordance index = 0.74.

After discharge from the hospital, patients were followed during preplanned visits after one, three, and six months. Events during follow-up were coded on the day of liver transplantation, death, or censored after more than 6 months. Survival status was verified in the national registry of deceased inhabitants. To clarify the effect of frailty on the prognosis in both groups, we constructed a Kaplan–Meier survival curve and performed a log-rank test (Figure 1). Furthermore, we used the Cox model to determine the relative hazard of death or LT during follow-up in NAFLD compared to ALD patients. For inherent differences in some prognostic variables between the study groups, we adjusted the model for age, sex, MELD score, C-reactive protein (CRP), HCC, and LFI (Figure 2). The results of the model with risk ratios (HR), 95% confidence intervals (CI), and *p* values are shown in the forest plot (Figure 3). To explore the sensitivity of the hazard ratio for death/LT to the rise of LFI, we added the hazard ratio of NAFLD/frailty to the model. This approach allowed us to quantify the difference in sensitivity to the rise in LFI between patients with NAFLD and ALD.

Statistical analysis has been carried out using the *R* software (R foundation for statistical computing, http://www.r-project.org), *R* Studio (v.1.2.5033, RStudio Inc. for macOS) with the EZR plugin, and MedCalc (MedCalc Software Ltd, Ostend, Belgium).

## 3. Results

From the registry which at the time included 1221 patients, we identified 385 eligible patients who met the entry criteria. Among them, 280 and 105 patients had alcoholic and NAFLD etiology, respectively. Patients with NAFLD were significantly older and had a higher proportion of females, higher BMI, MAC, and the triceps skinfold (Table 1). Functional parameters such as hand grip strength, chair stands per second, or balance time did not differ between the groups. The LFI was numerically lower in NAFLD patients, but the difference was not statistically significant. Also, NAFLD patients had better baseline parameters of synthetic liver function, MELD score, Child-Pugh-Turcotte score, and lower markers of systemic inflammation (white blood cells, CRP). NAFLD patients also had a higher proportion of cases with hepatocellular carcinoma (HCC, 17.1 vs. 6.8%, *p* = 0.002) and a lower proportion of refractory ascites.

Due to inherent sex-related differences in body composition, we also compared both groups according to sex (Table 2). No difference in nutritional parameters, inflammatory markers, or synthetic liver function between NAFLD and ALD in both sexes was observed. However, LFI was significantly lower in NAFLD men compared to ALD men, but we did not find similar differences in women.

We investigated potential risk factors for frailty separately for NAFLD and ALD in two models. In NAFLD patients, logistic regression yielded the following independent predictors of frailty (LFI > 4.5): male sex (OR = 0.31, 95% CI 0.12–0.816), BMI (OR = 1.16, 1.04–1.28), MAC (OR = 0.79, 0.68–0.91), and CRP (OR = 1.04, 1.01–1.06). In ALD patients, it was age (OR = 1.09, 1.05–1.12), male sex (OR = 0.47, 0.25–0.87), MELD score (OR = 1.11, 1.05–1.16), and the serum albumin (OR = 0.93, 0.89–0.98) (Table 3). The linear model yielded four independent LFI predictors throughout the patient cohort: age, sex, serum albumin, and the CRP. Besides, body mass index and MAC were other predictors of LFI in NAFLD patients and MELD scores in ALD patients (Table 4).

During follow-up, death or LT occurred within 30, 90, and 180 days in 14.3%, 26.8%, and 37.9% of ALD patients and 13.3%, 27.6%, and 35.2% of NAFLD patients, respectively, with no statistically significant differences between groups. Liver transplantation was carried out in 14 (5.0%) of ALD and 7 (6.7%) of NAFLD cases. The probability of transplant-free survival in both groups stratified according to frailty is displayed in Figure 1. The risk of death or LT was significantly higher in frail compared to nonfrail patients in both groups (*p* < 0.001). In the Cox model that predicts transplant-free survival after adjustment for age, sex, MELD, CRP, HCC, and LFI, NAFLD disease etiology was an independent predictor of death/LT (Figure 2, OR = 1.88 95% CI 1.32–2.67, *p* < 0.001). Forest plot with details of the model is displayed in Figure 3. The model also showed that the HR for death or LT for NAFLD etiology was more sensitive to the rise in LFI compared with ALD disease etiology (HR = 1.51, 1.05–2.2).

## 4. Discussion

Our study provides evidence that first, frailty substantially increases mortality in patients with cirrhosis of both etiologies. Second, the LFI retains its prognostic power with cutoffs validated in the original study [44, 45]. Third, NAFLD etiology increases the risk of death compared to ALD. Fourth, the impact of frailty on mortality appears to be stronger in NAFLD than in ALD patients.

Upon admission to the hospital, patients with NAFLD and ALD showed a similar prevalence of frailty, indicating a comparable susceptibility to incoming stressors. The observed differences in age, sex, and nutritional status between the groups reflected the differences in the natural history of the disease. In Central Europe with a high prevalence of cirrhosis [1, 46], the median age of ALD cirrhosis at its diagnosis is usually in the mid-fifties [47, 48]. In NAFLD cirrhosis, due to different pathogenetic factors, progression to cirrhosis appears to be slower [49]. Also, the NAFLD cirrhosis outbreak in Central Europe is delayed compared to Western Europe or the USA owing to the later adoption of the Western lifestyle and stronger cultural ties to alcohol. In the region, comprehensive data on the epidemiology and demography of NAFLD cirrhosis are still lacking. However, our data are compatible with some studies from other regions. Sanyal et al. reported a lower incidence of refractory ascites and lower MELD/CTP scores in 150 patients with nonalcoholic steatohepatitis (NASH) cirrhosis compared with HCV cirrhosis. Also, the rate of decompensation and cirrhosis progression was lower in NAFLD patients [50]. In contrast to other previously reported cohorts of NAFLD cirrhosis [51, 52], our study reports data from the registry of hospitalized patients with decompensated disease. In the literature, data on the outcome of decompensated NASH cirrhosis compared with ALD cirrhosis are scarce. One of the studies reported that once the cirrhosis decompensated, the overall survival and liver-related mortality were similar for both etiologies [53]. In the second study, authors reported lower liver-related mortality in NAFLD cirrhosis [54], but once the cirrhosis is decompensated, liver-related mortality was the leading cause of death.

An explanation for the principal findings may lie in the equation: frailty *×* burden = outcome. Since the prevalence of frailty was comparable, the difference in the outcome would imply the difference in the burden. Baseline characteristics in NAFLD patients show an additional five years in age and only partially reflect a higher disease burden. Although their liver disease burden was more favorable compared to ALD, they had a higher prevalence of HCC. In this study, however, only initial stages of HCC were included, and the presumed impact of HCC on mortality was not confirmed. Even though we adjusted our model for all known confounders, we did not adjust for all comorbid conditions, since our registry does not contain such data. Thus, it is conceivable that NAFLD etiology per se is a composite surrogate of the burden that metabolic syndrome with its extrahepatic manifestations implies on ACLD patients [55] and that baseline disease characteristics do not reflect the overall disease burden. Once frailty has arisen, it reflected a profound effect of the burden of all diseases: the liver-related burden and the burden of comorbid conditions. Similar findings have also resonated in some previous reports among LT candidates. Here, NAFLD patients were three times less likely to be listed for LT compared with patients with viral hepatitis, but they were more likely to die from their liver disease rather than their comorbid conditions [56, 57]. In our small volume liver transplantation center, the reduced chance of enrolling NAFLD patients on the waiting list has not been confirmed. However, a higher likelihood of dying from liver disease was compatible with our results (see limitations paragraph). In contrast, ALD patients initially present with a more pronounced systemic inflammation and jaundice. Once they begin to abstain, they receive treatment for alcoholic hepatitis and/or systemic antibiotics, and their condition usually improves substantially. Thus, their initial disease characteristics often overestimate the severity of their ALD [58].

Physical frailty assessment using LFI has proven to be a quick and easy tool suitable for the cirrhosis registry. The LFI independently predicted mortality in both cirrhosis etiologies. Our study thus supports in real-life the sustainability of this tool in the context of a resource-limited healthcare system. Our results also validate the diagnostic LFI cutoff of 4.5 in the population of nonwaitlisted patients while retaining its predictive value derived from the original study [44]. But in this study, contrary to our findings, waitlisted NAFLD patients had a higher prevalence of frailty compared to other etiologies [40]. One possible explanation would be in the timing of frailty investigations. Is it likely that ALD patients on the waiting list had recovered from the toxic effects of alcohol and its systemic inflammatory complications.

Our study explores different predictors of frailty in ALD and NAFLD cirrhosis. Age, sex, CRP, and albumin were identified as risk factors in both groups. Higher serum bilirubin concentrations in ALD drove the MELD score high and were likely related to recent alcohol consumption and alcoholic hepatitis. Alcohol has a profound toxic effect on muscle function [15], and once the consumption is stopped, muscle function may improve. In contrast, frailty in NAFLD patients was positively associated with BMI following the previously confirmed effect of obesity on muscle mass and function [10]. Besides, MAC and subcutaneous fat are established indicators of nutritional status. Thus, higher BMI and lower nutritional status appeared as additional factors exacerbating frailty in NAFLD. The quick reversibility of such conditions is currently questionable and should be subjected to further research. The role of subcutaneous fat, particularly in women, has been described as a stronger predictor of prognosis compared to muscle mass [22]. Although LFI calculation is adjusted for sex, females in our study had a higher risk of frailty. Hence, our data support the assessment and interpretation of body composition and functional status only according to sex. It is beyond the scope of this study to discuss sex-related issues, but it provides complementary data to previous studies on liver transplantation candidates [25–27, 32–35].

Our study has several strengths. Our direct comparison of the two most important etiologies of cirrhosis is rather unique. Due to the recent introduction of LFI as a tool for diagnosing physical frailty in cirrhosis, there is a paucity of data among hospitalized patients [59]. Our study has several limitations. RH7 registry data are limited by the lack of an exhaustive list of comorbidities. A relatively low number of NAFLD cases do not provide sufficient statistical power to address the impact of all such comorbidities. Contrary to our report, some previous studies reported liver-related mortality and not all-cause mortality. Thus, since liver-related mortality could only affect a subgroup of patients, the exact explanation of the increased all-cause mortality in NAFLD patients cannot be provided with confidence. However, when confronted with decisions on patients' management, all diseases need to be taken into account, and our study brings evidence that LFI appears to reflect that. Contrary to the previous studies addressing NAFLD etiology, we did not collect enough computed tomography (CT) results to enrich the muscle mass analysis as suggested by the EWGSOP2 guidelines. However, this limitation is not exceptional in the literature and highlights the advantage of the real-time availability of LFI in the daily practice of many healthcare settings.

## 5. Conclusions

Our study provides a unique insight into the differences between NAFLD and ALD cirrhosis in hospitalized patients with decompensated disease. Despite older age and a higher proportion of women, NAFLD patients showed a lower liver disease burden and a higher prevalence of HCC. Frailty was equally prevalent and drove all-cause mortality up in both groups. Age, female sex, serum albumin, and systemic inflammatory markers were risk factors for frailty in all patients. Besides, body mass index and MAC were other risk factors of frailty in NAFLD and MELD scores in ALD patients. Frailty and NAFLD demonstrated an independent effect on the risk of death or liver transplantation. Also, NAFLD patients compared to ALD had increased all-cause mortality. Having a higher sensitivity to frailty due to the overall disease burden and lower potential for improvement, management of frailty in NAFLD cirrhosis appears particularly challenging and requires an individualized approach. To improve the prognosis of these patients, we need more interventional studies with clinical endpoints.

## Figures and Tables

**Table 1:** Summary statistics and characteristics of the study groups, a comparison of NAFLD cirrhosis and alcoholic cirrhosis patients.

*N*	Group	Alcoholic cirrhosis	NAFLD cirrhosis	*p* value
		*N* = 280	*N* = 105	
Age, years		56.91 (48.56, 63.00)	62.26 (55.71, 67.13)	<0.001
Sex, *n* (%)	Female	85 (30.4)	49 (46.7)	0.004
	Male	195 (69.6)	56 (53.3)	
Body mass index, (kg/m^2^)		25.96 (23.06, 29.77)	28.63 (25.34, 34.88)	<0.001
Obese, *n* (%)		68 (24.3)	44 (41.9)	0.001
Mid-arm circumference (cm)		26.00 (23.00, 29.00)	29.00 (25.00, 33.00)	<0.001
Tricipital skinfold (mm)		9.00 (6.20, 15.10)	14.00 (7.80, 21.40)	<0.001
Mid-arm muscle area (cm^2^)		40.23 (33.01, 48.31)	43.92 (35.25, 57.90)	0.002
Hand grip strength (kg)		22.55 (15.88, 29.85)	23.13 (15.83, 29.86)	0.945
Low hand grip strength, *n* (%)		198 (70.7)	67 (63.8)	0.217
Chair stands (s)		0.36 (0.27, 0.43)	0.39 (0.30, 0.48)	0.167
Chair stands categories, *n* (%)	Normal	23 (8.2)	16 (15.2)	0.133
	Low	159 (56.8)	57 (54.3)	
	Unable to stand	98 (35.0)	32 (30.5)	
Equilibrum total time (s)		30.00 (20.00, 30.00)	30.00 (24.58, 30.00)	0.143
Equilibrum categories, *n* (%)	Normal	165 (58.9)	68 (64.8)	0.498
	Low	77 (27.5)	27 (25.7)	
	Unable to stand	38 (13.6)	10 (9.5)	
Liver frailty index (LFI)		4.48 (3.97, 5.04)	4.28 (3.81, 4.87)	0.061
Frailty, LFI > 80^th^ percentile, *n* (%)		60 (21.4)	14 (13.3)	0.082
Frailty, LFI > 4, 5		134 (47.9)	50 (47.6)	1.00
Serum bilirubin (umol/l)		50.0 (26.51, 137.05)	26.2 (18.8, 76.75)	<0.001
Serum albumin (g/l)		28.90 (24.00, 33.00)	29.00 (27.00, 35.00)	0.018
Serum creatinine (umol/l)		77.90 (59.00, 113.00)	79.00 (63.00, 113.00)	0.868
C-reactive protein (mg/l)		16.23 (6.94, 40.20)	10.84 (4.99, 22.79)	0.010
White blood cells (*∗*10^9^/l)		7.20 (4.88, 11.03)	5.80 (3.80, 7.50)	<0.001
MELD-Na score		18.91 (14.00, 24.00)	15.00 (11.00, 19.00)	<0.001
Child-Pugh-Turcotte score		10.00 (7.00, 11.00)	8.00 (7.00, 10.00)	<0.001
Hepatocellular carcinoma, *n* (%)		19 (6.8)	18 (17.1)	0.002
Refractory ascites, *n* (%)		89 (33.1)	23 (22.3)	0.044
Number connection test, *n* (%)	Normal	37 (13.2)	13 (12.4)	0.778
	60–90	69 (24.6)	28 (26.7)	
	90–120	70 (25.0)	29 (27.6)	
	>120	83 (29.6)	25 (23.8)	
	Not done	21 (7.5)	10 (9.5)	
Event during follow-up, *n* (%)	None, LT, death	145, 14, 121 (51.8, 5.0, 43.2)	52, 7, 46 (49.5, 6.7, 43.8)	0.771
Mortality	30 days	40 (14.3)	14 (13.3)	0.87
	90 days	75 (26.8)	29 (27.6)	0.898
	180 days	106 (37.9)	37 (35.2)	0.723

**Table 2:** Sex-specific summary statistics and characteristics of the study groups, comparison of alcoholic and NAFLD cirrhosis.

*n*	Group	Men		*p* value	Women		*p* value
		Alcoholic cirrhosis	NAFLD cirrhosis		Alcoholic cirrhosis	NAFLD cirrhosis	
		*N* = 195	*N* = 56		*N* = 85	*N* = 49	
Age, years		58.00 (48.88, 63.03)	62.47 (57.50, 67.14)	0.002	55.36 (47.47, 61.27)	61.97 (53.91, 67.00)	0.006
Body mass index (kg/m^2^)		27.04 (23.54, 30.41)	29.54 (26.62, 33.58)	0.001	24.24 (21.87, 27.90)	26.78 (24.61, 35.75)	<0.001
Obese, *n* (%)		54 (27.7)	24 (42.9)		14 (16.5)	20 (40.8)	0.003
Mid-arm circumference (cm)		26.50 (24.00, 30.00)	29.00 (26.75, 33.00)	<0.001	24.00 (22.00, 27.00)	27.00 (24.00, 30.00)	<0.001
Tricipital skinfold (mm)		9.00 (6.00, 14.00)	12.20 (7.15, 18.80)	0.016	11.00 (7.00, 17.00)	17.20 (9.40, 24.00)	0.005
Mid-arm muscle area (cm^2^)		41.79 (35.67, 50.44)	51.18 (40.69, 63.06)	0.001	34.16 (27.37, 42.50)	39.55 (32.94, 49.88)	0.006
Hand grip strength (kg)		26.27 (21.37, 32.50)	29.07 (24.71, 34.39)	0.018	14.83 (11.07, 17.83)	17.03 (12.43, 19.93)	0.194
Low hand grip strength, *n* (%)		127 (65.1)	30 (53.6)	0.12	71 (83.5)	37 (75.5)	0.267
Chair stands/s		0.36 (0.28, 0.43)	0.44 (0.35, 0.51)	0.004	0.35 (0.26, 0.42)	0.30 (0.22, 0.41)	0.398
Chair stands categories, *n* (%)	Normal	18 (9.2)	12 (21.4)	0.044	5 (5.9)	4 (8.2)	0.881
	Low	110 (56.4)	30 (53.6)		49 (57.6)	27 (55.1)	
	Unable to stand	67 (34.4)	14 (25.0)		31 (36.5)	18 (36.7)	
Equilibrum total time (s)		30.00 (20.00, 30.00)	30.00 (27.07, 30.00)	0.077	30.00 (13.00, 30.00)	30.00 (20.00, 30.00)	0.448
Equilibrum categories, *n* (%)	Normal	118 (60.5)	40 (71.4)	0.353	47 (55.3)	28 (57.1)	0.607
	Low	55 (28.2)	12 (21.4)		22 (25.9)	15 (30.6)	
	Unable to stand	22 (11.3)	4 (7.1)		16 (18.8)	6 (12.2)	
Liver frailty index (LFI)		4.37 (3.91, 4.95)	4.05 (3.59, 4.68)	0.004	4.67 (4.15, 5.40)	4.65 (4.03, 4.96)	0.590
LFI > 80^th^ percentile = 5.2, *n* (%)		35 (17.9)	5 (8.9)	0.146	25 (29.4)	9 (18.4)	0.216
LFI > 4.5		87 (44.6)	18 (32.1)	0.124	47 (55.3)	32 (65.3)	0.279
Serum bilirubin (umol/l)		46.8 (24.8, 113.7)	25.0 (19.9, 46.7)	0.001	75.5 (29.3, 164.9)	27.2 (18.3, 124.4)	0.01
Serum albumin (g/l)		28.00 (24.61, 32.00)	31.00 (26.75, 37.25)	0.002	29.00 (24.00, 34.00)	28.90 (27.00, 32.00)	0.989
Serum creatinine (umol/l)		81.00 (62.00, 119.00)	83.00 (66.75, 118.25)	0.433	74.00 (54.00, 99.00)	71.00 (54.00, 94.10)	0.948
C-reactive protein (mg/l)		14.52 (6.83, 34.20)	8.73 (4.16, 17.75)	0.010	25.29 (7.16, 50.33)	14.61 (6.28, 29.14)	0.122
White blood cells (∗10^9^/l)		6.80 (4.80, 10.60)	5.50 (4.00, 7.32)	0.001	8.00 (5.40, 12.56)	6.50 (3.70, 8.10)	0.003
MELD-Na score		18.89 (13.73, 24.00)	14.50 (11.00, 18.50)	<0.001	19.00 (14.00, 25.00)	15.00 (11.00, 20.00)	0.010
Child-Pugh-Turcotte score		9.00 (7.00, 11.00)	8.00 (6.00, 9.00)	<0.001	10.00 (8.00, 11.00)	9.00 (7.00, 10.00)	0.045
Refractory ascites, *n* (%)		68 (36.4)	13 (23.2)	0.076	21 (25.6)	10 (21.3)	0.671
Event during follow-up, *n* (%)	None, LT, death	95, 5, 22 (48.7, 5.1, 46.2)	29, 5, 22 (51.8, 8.9, 39.3)	0.413	50, 4, 31 (58.8, 4.7, 36.5)	23, 2, 24 (46.9, 4.1, 49.0)	0.383

**Table 3:** Predictive factors of frailty defined by the liver frailty index > 4.5, a multivariate logistic model.

Comparison of NAFLD cirrhosis and alcoholic cirrhosis			
	OR	95% CI	*p* value
Nonalcoholic fatty liver cirrhosis			
Male sex	0.31	0.118–0.816	0.02
Body mass index	1.16	1.04–1.28	0.006
Mid-arm circumference	0.79	0.684–0.907	0.001
C-reactive protein	1.04	1.01–1.06	0.011
AUROC = 0.85; 95% CI 0.773–0.928			

Alcoholic cirrhosis			
Age	1.09	1.05–1.12	<0.001
Male sex	0.47	0.25–0.867	0.016
MELD	1.11	1.05–1.16	<0.001
Albumin	0.93	0.891–0.984	0.01
AUROC = 0.763; 95% CI 0.707–0.819			

Variables in the model: albumin, BMI, tricipital skinfold, serum creatinine mid-arm circumference, male sex, refractory ascites, age, MELD, and CRP.

**Table 4:** Predictive factors of the liver frailty index in a linear model.

Comparison of NAFLD cirrhosis and alcoholic cirrhosis				
	Estimate	Std. error	*t* value	*p* value
Nonalcoholic fatty liver cirrhosis				
Intercept	5.12	0.656	7.801	<0.001
Age	0.019	0.006	3.134	0.002
Sex, male	−0.27	0.130	−2.08	0.04
Serum albumin, g/l	−0.041	0.011	−3.664	<0.001
C-reactive protein, mg/l	0.006	0.001	6.609	<0.001
Body mass index	0.024	0.011	2.238	0.028
Mid-arm circumference, cm	−0.046	0.017	−2.694	0.008
Multiple R-squared: 0.5034	Adjusted R-squared: 0.4494			
*F*-statistic: 9.326 on 10 and 92 DF, *p* value: 1.671e-10				
Alcoholic cirrhosis				
Intercept	3.433	0.445	7.707	<0.001
Age	0.026	0.004	5.948	<0.001
Sex, male	−0.326	0.101	−3.206	0.01
Serum albumin, g/l	−0.025	0.008	−3.068	0.002
C-reactive protein, mg/l	0.004	0.001	3.096	0.002
MELD score	0.027	0.007	3.580	<0.001
Multiple R-squared: 0.2756,	Adjusted R-squared: 0.2476			
F-statistic: 9.818 on 10 and 258 DF, *p* value: 6.7e-14				

Variables in the model: albumin, BMI, tricipital skinfold, serum creatinine. mid-arm circumference, male sex, refractory ascites, age, MELD, and CRP.

## Data Availability

The data from the RH7 registry used to support the findings of this study are owned by the Department of Hepatology, Gastroenterology, and Transplantation and will be available on the Mendeley Data repository. The link will be provided from the corresponding author upon request.
